# Impact of digitalization-related STEM in-service teacher trainings in cooperation with out-of-school student labs on teachers’ professional knowledge, self-efficacy and technology commitment

**DOI:** 10.3389/fpsyg.2025.1656648

**Published:** 2025-11-12

**Authors:** Anna Reher, Mathias Ziegler, Eva Blumberg, Mahdi El Tegani, Colin Peperkorn, Kerstin Röllke, Stefanie Schwedler, Lisa Stinken-Rösner, Janne Lene Wassing, Tim Kirchhoff

**Affiliations:** 1 Faculty of Mathematics, Institute for Didactics of Mathematics (IDM), Bielefeld University, Bielefeld, Germany; 2 Faculty of Physics, Physics Education, Bielefeld University, Bielefeld, Germany; 3 Faculty of Science, Department of Physics, Paderborn University, Paderborn, Germany; 4 Faculty of Biology, Biology Didactics - Giftedness and Talent Research, Bielefeld University, Bielefeld, Germany; 5 Faculty of Biology, Didactics of Biology, Bielefeld University, Bielefeld, Germany; 6 Faculty of Chemistry, Chemistry Education, Bielefeld University, Bielefeld, Germany; 7 Faculty of Biology, Primary Science Education, Bielefeld University, Bielefeld, Germany

**Keywords:** in-service teacher training, STEM, out-of-school student lab, digitalization, TPACK, self-efficacy, technology commitment

## Abstract

**Introduction:**

Increasing digitalization in school expands teachers’ options to design learning arrangements, but also creates demand to promote digitalization-related skills in STEM disciplines. Given this growing demand, universities are being asked to participate in the design and evaluation of evidence-based in-service teacher trainings. University-based out-of-school student labs have already demonstrated capacity to develop innovative offerings related to domain-specific digital technologies. Their incorporation into pre-service teacher trainings has yielded favorable outcomes. Consequently, there is potential to incorporate them into in-service teacher training as well. Research is needed regarding the impact of such trainings at different levels, particularly with respect to teachers’ professional knowledge and beliefs.

**Methods:**

In this study, eight different in-service teacher trainings covering various STEM disciplines were designed in cooperation with out-of-school student labs to promote the use of subject-specific media in the classroom. We examined the overall effects of these trainings on teachers’ beliefs regarding their professional knowledge, self-efficacy, and technology commitment. One hundred and five STEM-teachers (*M*_age_ = 41.74 years, *SD*_age_ = 9.45 years, 33% female) participated in eight distinct and discipline-specific in-service teacher trainings. Participants’ self-assessed professional knowledge, self-efficacy, and technology commitment were evaluated in a pre-posttest-design, using questionnaires with a 6-point Likert scale. Furthermore, outcomes and initial values of the intervention group of teachers were compared to those of a comparison group (*n* = 569; *M*_age_ = 40.92 years, *SD*_age_ = 11.46 years, 67% female).

**Results:**

The results of the pre-post comparison indicate a positive effect on all analyzed dimensions of self-assessed professional knowledge. No significant differences were found regarding participants’ self-efficacy and technology commitment. Additionally, participants’ prior technology commitment was already high and remained consistently high after intervention. This is confirmed by the results observed in the comparison group. The pretest values of the intervention group were significantly higher than those of the comparison group in the dimensions of self-assessed professional knowledge, technology commitment, and self-efficacy. Similar results were observed when posttest data of the intervention group were compared with those of the comparison subgroup comprising participants who had previously participated in other digitalization-related teacher trainings.

## 1 Introduction

In the context of digital transformation, the use of digital media has become an important aspect of school teaching [Kultusministerkonferenz (KMK), 2016; Organisation for Economic Co-operation and Development (OECD), 2015]. While these media offer innovative and powerful approaches to fostering learning and may enhance the overall quality of teaching, their variety, versatility, and complexity also impose new demands on teacher professionalization (Caena and Redecker, 2019). Therefore, in addition to fundamental pedagogic competencies, digitalization-related skills are necessary for teachers to use digital media pedagogically meaningful in their teaching practice (Fernández-Batanero et al., 2020). This applies particularly to domain-specific digital media in STEM subjects. In this regard, domain-specific digital media, such as (interactive) experimental videos or virtual labs, open new possible applications in STEM lessons (Weiler et al., 2023) and promote individual learning by enabling participation of all learners in the respective subject lessons (Abels and Stinken-Rösner, 2022). Although there has been a significant increase in the use of digital media during the last twelve years (Drossel et al., 2024), this mainly relates to established and cross-disciplinary media (such as software of presentation). More innovative and domain-specific media (such as simulations or interactive experimental videos) are still rarely used in classrooms (Drossel et al., 2024; Vogelsang et al., 2019). In order to exploit the potential of these media in STEM lessons in a pedagogically meaningful way, teachers must apply both general digitalization-related skills and domain-specific skills as described in the Digital Competencies for Science Education (DiKoLan) model, for example (Becker et al., 2020). However, prior studies revealed teachers to dispose of rather rudimentary digitalization-related skills (Huber et al., 2020; Fernández-Batanero et al., 2020). Despite the availability of teacher trainings (from now on referred to as TT) addressing this issue, teachers partook only to a limited extent (Eickelmann, 2019). According to the offer-and-use model (Lipowsky and Rzejak, 2019), it should be noted that more TTs offers do not necessarily lead to skill development. The important factors are whether and how these offers are perceived by teachers as well as how the training contents are actively used and transferred to teachers’ domain-specific teaching. A lack of adequate domain-specific programs is one reason for non-participation in TTs [Diepolder et al., 2021; GEW (Gewerkschaft Erziehung und Wissenschaft), 2020]. This is also reflected by teachers’ expressed needs for professionalization (Drossel et al., 2024). Therefore, further domain-specific in-service TTs are necessary to foster teachers’ digitalization-related domain-specific skills concerning the implementation of technology in STEM-teaching.

Out-of-school student labs as extracurricular learning environments are especially well-suited to demonstrate possible applications of domain-specific media in a school-like setting while also providing a protected and assisted training environment for teachers (Haupt, 2023). However, little is known on suitable formats for such TTs in cooperation with out-of-school labs as well as their possible impacts on teachers’ beliefs and digitalization-related skills. Considering this, within the joint project “Student labs as places for advanced teacher training in the digital world” (LFB-Labs-digital), eight domain-specific TTs in cooperation with student labs as an environment for in-service TTs (Kirchhoff et al., 2024) have been developed and evaluated. These aim to expand the professional knowledge of teachers, impart their digitalization-related domain-specific skills and foster self-efficacy as well as technology commitment.

## 2 Theory and empirical evidence

**Figure 1:**
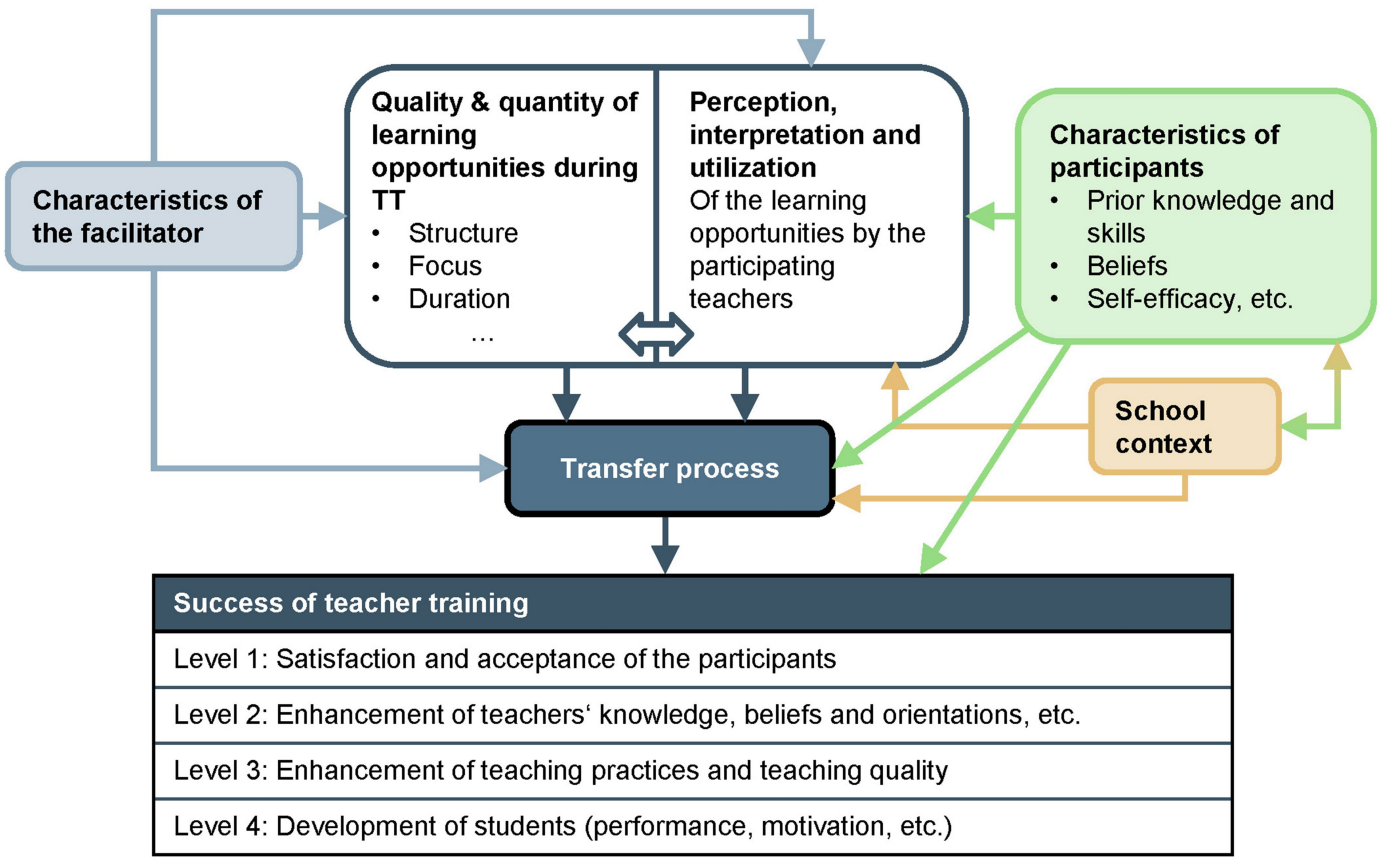
Offer-and-use model for research on teacher training [according to Lipowsky and Rzejak (2019)].

The success of a TT depends on numerous factors that go beyond the high-quality design of the training itself. According to the offer-and-use model proposed by Lipowsky and Rzejak (2019) (see Figure 1), it is not only the characteristics of the facilitator, the prerequisites of the participating teachers, and the school context that are key factors. In particular, the quality and quantity of learning opportunities during TT as well as the perception and use by the participating teachers potentially influence the transfer process into practice.

Success of the training itself results from the transfer process initiated as well as individual prerequisites of participating teachers. It can be described on four levels: (1) Acceptance and satisfaction of the participating teachers, (2) expansion of teacher knowledge, further development of beliefs and orientations, etc. (3), further development of teaching quality, and (4) promotion of student learning. The present study utilizes level 2 evaluations to assess the success of the TTs, as all TTs were designed to address level 2 and quantitative data were collected as part of the project. Furthermore, a qualitative investigation was conducted to assess individual TTs at levels 3 and 4. However, these aspects are not considered in this study. As illustrated in Figure 1, the participants’ characteristics have an impact on the level of success achieved through the TTs. At level 2 the expansion of the teachers’ beliefs, including self-assessed knowledge based on professional knowledge (TPACK framework), self-efficacy, and technology commitment, are measured using validated questionnaires.

### 2.1 The TPACK framework

Successful implementation of digital media in the classroom is largely dependent on teachers’ expertise, which includes the ability to use digital media appropriately to impart specialized knowledge. To design effective TTs, previous studies, aligned with Lipowsky and Rzejak (2019), highlight the importance of integrating various facets of professional knowledge related to digitalization (e.g., Cabero and Barroso, 2016; Rodríguez Moreno et al., 2019).

According to Mishra and Koehler (2006), the TPACK framework describes teachers’ professional knowledge needed to implement digital media in the classroom. Its core idea is to integratively combine three dimensions of central knowledge: Technological, Pedagogical, and Content Knowledge. Pedagogical knowledge (PK) encompasses the educational knowledge about methods of teaching regardless of the subject, while the content knowledge (CK) contains content-related specialized knowledge about the actual subject content that is to be taught. The third dimension of technological knowledge (TK) deals with technical aspects, such as the use of hardware and software like tablets or writing programs in the classroom.

**Figure 2:**
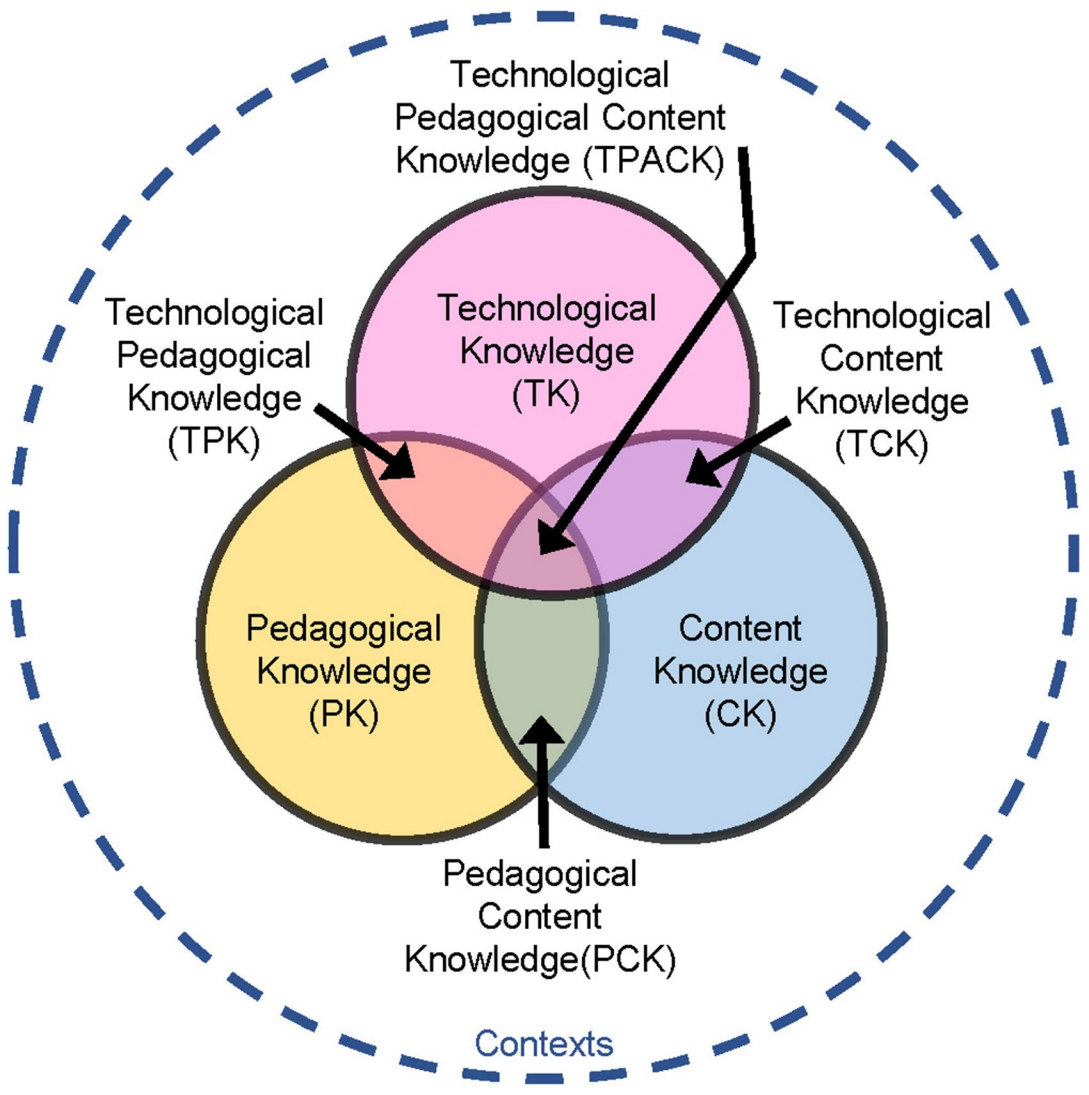
TPACK framework [according to Koehler and Mishra (2009)].

These knowledge dimensions are not considered separate from each other but are regarded as overlapping areas, thereby forming four intersecting knowledge areas (see Figure 2). Pedagogical content knowledge (PCK) is equivalent to domain-specific educational knowledge and describes how content-related specialized knowledge can be comprehensible to students (e.g., knowledge about the handling of learning difficulties in domain-specific instruction). Technological content knowledge (TCK) represents the knowledge that results from the reciprocal relationship between technology and content. Teachers must not only master the subject content, but also understand which technologies are best suited for learning specialized contents (Koehler and Mishra, 2009; e.g., knowledge about the use of software specifically designed for domain-specific instruction). The dimension of technological pedagogical knowledge (TPK) describes the understanding of how digital media can be used in a didactically way to support teaching regardless of the subject (e.g., knowledge about the benefits of digital media for illustrating everyday examples to students).

Technological pedagogical content knowledge (TPACK) is the central component of the TPACK framework and describes the knowledge for teaching content-related subjects using digital media (e.g., knowledge about the design of learning environments in which subject-specific content, pedagogical principles, and digital media are appropriately integrated).

Recent studies on self-assessed professional knowledge interpret the TPACK framework as transformative, whereby the fundamental knowledge dimensions (CK, PK, and TK) predict the primary hybrids (TPK, TCK, and PCK), which subsequently predict TPACK (Stinken-Rösner, 2021; Angeli and Valanides, 2005; Jang and Chen, 2010; Jin, 2019; Schmid et al., 2020). It has also been shown that the TPK and PCK dimensions significantly influence TPACK (with TPK having the highest impact), whereas the TCK dimension does not (Stinken-Rösner, 2021; Schmid et al., 2020). Therefore, the pedagogical-technological (TPK) and pedagogical-content (PCK) components in particular play a central role in the development of comprehensive technological-pedagogical-content knowledge (TPACK) and should be addressed in TTs. The technological-content dimension (TCK), on the other hand, appears to be of lesser importance.

Furthermore, current research indicates that targeted interventions can effectively foster self-assessed TPACK among pre-service STEM teachers. Specific courses within teacher education programs have been shown to significantly improve pre-service STEM teachers’ TPACK self-assessment (Stinken-Rösner et al., 2023; Chai et al., 2010; Max et al., 2024). In-service STEM teachers’ self-assessed TPACK can also be promoted through specific TT (Lehiste, 2015) as well as online courses (Rientjes et al., 2013). Finally, TPACK constitutes the main area of knowledge that teachers need to apply digital media in a pedagogically meaningful and content-appropriate way. The TPACK framework therefore provides a theoretical foundation for designing effective in-service TTs that target teachers’ digitalization-related professional knowledge.

### 2.2 Self-efficacy

Beside possessing suitable professional knowledge, teachers need to incorporate this knowledge into teaching practice (Stinken-Rösner et al., 2023). In this respect, it is fundamental to consider individual beliefs, such as teachers’ self-efficacy and their technology commitment. For example, self-efficacy correlates with the integration of digital media into teaching practices (Clipa et al., 2023). It can thus be concluded that the promotion of these factors has the potential to encourage their implementation in the classroom.

Self-efficacy in general is defined as the subjective conviction of “one’s own competence to cope with new or difficult challenging situations” (translated from Schwarzer and Jerusalem, 2002, p. 35). Regarding digitalization in teaching, self-efficacy in dealing with digital media refers to the belief that one can use computers and other digital technologies competently (Spannagel and Bescherer, 2009). It is an important factor for the successful use and integration of digital media in an educational context. Self-efficacy determines whether a certain behavior, such as the use of domain-specific media, is initiated (Bandura, 1977). Furthermore, the degree of effort invested in carrying out the action, despite possible obstacles, depends on self-efficacy (Bandura, 1977). Teachers who have a high level of confidence in their ability to use digital media are more likely to experiment with new technologies in the classroom and less likely to give up when problems arise (Schweizer and Horn, 2014). Therefore, an increased self-efficacy is a positive factor influencing the implementation of digital media in the classroom and thus represents a construct that should be promoted through TTs.

A meta-analysis by Zhou et al. (2023) examined the effectiveness of further trainings for STEM teachers to improve self-efficacy. A total of 21 studies with 48 independent cases from the years 1997 to 2022 were analyzed. The authors concluded that participation in further trainings for STEM teachers has a statistically significant influence on their self-efficacy. A medium effect size was indicated (Zhou et al., 2023). Further studies confirm this finding (e.g., Liu and Liao, 2019; Yang, 2020). In agreement with Liu and Liao (2019) and Yang (2020), Zhou et al. (2023) also state that the scope (in hours) of training has an influence on the improvement of teachers’ self-efficacy. However, Zhou et al. emphasize that this effect only occurs for teachers who have taken part in domain-specific further training. No information was provided whether digitalization-related TT was also considered in the study. Digitalization-related TTs that aim to promote self-efficacy of STEM-teachers using technology are still rare. Thurm and Barzel (2020) analyzed self-efficacy in the context of using technology before and after participating in a digitalization-related TT. The results of this study show an increase in self-efficacy when teaching with digital media. Compared to the control group, whose teachers did not take part in a training but also showed increased self-efficacy, the observed effect could not ultimately be attributed to participation in the training. Thurm and Barzel (2020) therefore call for TTs to place a stronger focus on promoting self-efficacy. This is realized in the trainings on which this study is based (see section 3).

### 2.3 Technology commitment

Within the framework of the offer-and-use model, teachers’ beliefs are a central prerequisite for the implementation of digital media, of which the technology commitment (Neyer et al., 2012) is a relevant factor. The construct comprises three dimensions: Technology acceptance (based on Davis et al., 1989), technology competence beliefs and technology control beliefs (both based on Krampen, 1991). According to Neyer et al. (2012), the term technology acceptance refers to the subjective evaluation of technological advances by a particular person. Technology competence beliefs are defined as a subjective assessment of one’s own abilities, which includes both the experience already gained with familiar media and the ability to adapt to innovative media. They thus represent a sub-area of self-efficacy (see section 2.2). Finally, technology control beliefs are defined as the extent to which a particular person perceives influence or control over technical processes. Additionally, Neyer et al. (2012) state that there is a stronger correlation between the use of technology and the dimensions of technology acceptance and technology competence beliefs than with technology control beliefs. We therefore limit ourselves to these two dimensions in this study. A high level of technology commitment promotes the implementation of digital media in the classroom. Hence, teachers are more likely to use digital media if they have a positive attitude towards them (Davis et al., 1989) As a result, there is a need for TTs, which strengthen both the necessary digitalization-related skills of the teachers and promote their technology commitment. Current studies point to promising approaches for pre-service teachers (Köstler and Wolff, 2025).

### 2.4 Characteristics of effective in-service teacher trainings

Lipowsky and Rzejak (2021) emphasize that effective TTs should offer domain-specific content and pedagogical strategies, a view supported by research highlighting the importance of such tailored programs for STEM teachers to equip them with the necessary knowledge and instructional strategies (Kowalski et al., 2020). In this context, adaptability is essential. Wilson et al. (2025) underscore the importance of delivering content that aligns with immediate requirements, particularly given time constraints, and the challenges of integrating innovations into the classroom while adhering to established curricular standards. Building on this, the aspect of integrating input, implementation, and reflection phases is crucial, as it allows teachers to test their newly acquired knowledge, reflect on their experiences, and facilitate its transfer into teaching practice (Lipowsky and Rzejak, 2019). This has also been confirmed in STEM education, where long-term support during TTs is essential (Wilson et al., 2025). TTs with an extended duration are more effective in terms of teachers attitudes and classroom practice than one-time interventions (Scher and O’Reilly, 2009) or short-term TTs lasting fewer than 3 weeks (Kowalski et al., 2020).

A fundamental aspect of digitalization-related trainings is the enhancement of digital teaching practices when the use of digital tools extends beyond basic technical handling to include a focus on instructional content (Koschorreck and Gundermann, 2020; Runge et al., 2024). Cendon (2018) highlights the need to integrate the perspectives of both instructors and learners in training programs. In connected learning environments, the growing integration of digital technologies blurs the boundaries between learners and educators, emphasizing the collaborative nature of teaching and learning.

To integrate these insights within the framework of evidence-based and practically applicable design and evaluation of TTs, it is both conceivable and necessary to institutionalize the involvement of universities in the context of in-service TTs (Riecke-Baulecke, 2023). To incorporate domain-specific and digitalization-related content into TTs, it is essential to strengthen domain-specific educational disciplines as interfaces between pedagogical content knowledge (PCK) and technological content knowledge (TCK) in professional development (Riecke-Baulecke, 2023; Daschner et al., 2023). Hence, out-of-school, university-based environments, such as student laboratories, offer potential touchpoints for development as innovative sites for TTs (Kirchhoff et al., 2024).

### 2.5 Out-of-school student labs

Out-of-school student labs, also termed outreach science labs (hereafter referred to as student labs), are extracurricular learning environments that have been primarily established in STEM education and, increasingly, in the humanities and social sciences across Europe (Haupt, 2023). Key objectives include fostering interest and understanding of science and providing opportunities for engagement with current topics in STEM disciplines (Dähnhardt et al., 2009). A main focus is placed on experimentation and inquiry-based learning (Haupt, 2023). Student labs differ in terms of their sponsorship, specific objectives, and offerings. Most student labs are located at universities and universities of applied sciences (Haupt, 2023) and are often operated by institutions focused on domain-specific education (Haupt et al., 2013).

Classical student labs offer experimental days for whole classes or courses as part of school events, which are related to the current school curriculum (Scharfenberg et al., 2019). At the student level, visits have a positive impact on situational interest (Kirchhoff et al., 2023b; Schüttler et al., 2021) and intrinsic motivation (Kirchhoff et al., 2023a). Student labs that are actively involved in pre-service TTs are also known as teaching-learning labs. In these settings, pre-service teachers can gain practical teaching experience, apply pedagogical concepts, and plan lessons independently. Direct interaction with school students offers valuable insights into real-world teaching practices (Heß and Kratzer, 2023). Research findings in this context provide initial evidence of positive effects within pre-service TTs (Euler and Schüttler, 2020), including the initiation of pedagogical content knowledge (PCK) for students (Hume, 2012; Scharfenberg and Bogner, 2016; Rehfeldt et al., 2020).

In addition, student labs have the capacity to align with practical needs and swiftly develop offerings related to current digital technologies through close contact with schools. They have demonstrated a capacity for agile adaptation to emerging trends in digital content and formats over recent years (Haupt and Stamer, 2023). In addition to digital guides and survey instruments, simulations, animations, and subject-specific digital tools are also employed (Haupt and Stamer, 2023). In comparison to state educational institutions, they were able to face the challenges of the digital transformation more quickly and productively (Euler and Schüttler, 2020).

In order to ensure that these potential benefits of student labs and their resulting effects are sustainably integrated into the school environment, it is recommended to strengthen the network between student labs and the school system (Scharfenberg et al., 2019). To that end student labs might be expanded to centers for TT (Kirchhoff et al., 2024; Brüning et al., 2020). While numerous student labs already report offering (digitalization-related) TTs (Siegmund-Jürgens et al., 2023; Scharfenberg and Bogner, 2015), evaluation studies in this area, such as Hausamann (2012), remain rare. Further research is needed regarding the effectiveness of TT in cooperation with student labs, particularly in light of current training needs and evidence-based training design.

## 3 Research questions and hypotheses

It is a major aim of the project “LFB-Labs-digital” to design domain-specific in-service TTs on digital media in cooperation with out-of-school student labs, thereby spanning most STEM-disciplines. This study investigated the impact of eight TTs on teachers’ self-assessed professional knowledge, self-efficacy, and technology commitment. To examine the results in a broader context, the findings are compared with results of a comparison group. Specifically, we pursued the following research questions (RQ):

RQ1: Does participating in the TT enhance teachers’ self-assessed professional knowledge on applying digital media as framed by TPACK, and does it lead to stronger self-efficacy and greater technology commitment in STEM education?

RQ2.1: Do teachers who participate in the TT have greater self-assessed professional knowledge on applying digital media as framed by TPACK and do they have stronger self-efficacy regarding teaching with technology and a greater technology commitment before the TT than teachers who did not participate?

RQ2.2: Do teachers who participated in the TT have greater self-assessed professional knowledge on applying digital media as framed by TPACK and do they have stronger self-efficacy regarding teaching with technology and a greater technology commitment after the TT than teachers who participated in other digitalization-related TTs?

The following section outlines hypotheses for the research questions.

The design of the TTs is fundamentally based on evidence-based characteristics of effective in-service TT (see section 2.4). The integration of input phases that address domain-specific educational content and digital teaching practices is intended to expand the participants’ professional knowledge, with particular emphasis on PCK, TCK and TPACK as key knowledge dimensions. Moreover, implementation and reflection phases provide participants with the opportunity to apply the content throughout the training while receiving ongoing support. By adhering to these established characteristics, the TTs aim to initiate transfer processes that, according to Lipowsky and Rzejak (2019), can be measured in terms of improvements in teachers’ knowledge, beliefs, and related outcomes.

*Hypothesis* 1 (RQ1): We hypothesize that participating in the designed TTs is resulting in an increase in self-assessed professional knowledge, self-efficacy and technology commitment. With regard to the dimensions of professional knowledge the greatest increase is expected in the areas PCK, TCK and TPACK.

Given the absence of a quantifiable in-service TT obligation in most german states, including North Rhine-Westphalia (Daschner, 2023), it can be deduced that the participants enrolled in the designed TTs did so on their own initiative. Therefore, it can be assumed that participants exhibited a higher degree of involvement in the use of digital media in comparison to teachers who did not participate in the designed TTs.

*Hypothesis* 2.1 (RQ2.1): We hypothesize that participating teachers already possess greater self-assessed knowledge, stronger self-efficacy and a higher level of technology commitment than teachers who did not participate in the designed TT.

Knowing that the participants in the comparison group have previously engaged in other digitalization-related TT over the past 2 years before the conduction of this study, and it is conceivable that they have also participated in (domain-specific) TTs designed according to recent characteristics of effective TTs, the following hypothesis can be outlined.

*Hypothesis* 2.2 (RQ2.2): We hypothesize that the participants have a similar level of self-assessed professional knowledge, self-efficacy, and technology commitment after participating in the designed TTs as teachers who participated in other TTs related to digitalization.

## 4 Materials and methods

In this section, the designed TTs in cooperation with the student labs are described first, followed by the samples being characterized, a detailed description of the data collection process, the instruments used, and the analyses conducted.

### 4.1 Material design: teacher trainings in cooperation with out-of-school student labs

In line with the characteristics of effective in-service TT (see section 2.4), eight domain-specific in-service TTs were designed in cooperation with eight out-of-school student labs (Kiel and Schwedler, 2024; Kirchhoff et al., 2024; Lehmenkühler et al., 2024; Panhorst et al., 2025; Reher, n. d.; Ziegler and Stinken-Rösner, 2024). The TTs cover a variety of subjects, including Mathematics, Biology (with specific focuses on robotics and biotechnology), Chemistry, Physics, and Science Education. Each TT addresses domain-specific technology, such as simulations, (interactive) experimenting videos, phylogenetic software, and other applications. The training modules combine theoretical components with practical elements and reflection phases. While all training courses aim to enhance teaching practices through practical application, they differ in terms of their structure, duration and the specifics of their practical phases. Key features of the TTs are shown in Table 1. Initially, all trainings were planned to include an implementation phase in which materials from the student lab or material developed by the participants is tested during training in order to foster transfer. Testing takes place either in the classroom or in the student lab. Unfortunately, due to low enrollment numbers for the originally planned modular training courses, two of the trainings have been rescheduled as one-day courses, each consisting of a single module without an implementation phase (*n*_TT2/3_ = 10). The duration of the individual training sessions varies since these TTs were conducted multiple times.

**Table 1:** Key features overview of the developed teacher trainings (TT): subject, target group, basic structure, scope, duration, cooperation with the student lab, and number of participants.

TT	Subject	Target group^a^	Basic structure and scope in hours (h)	Duration in days/weeks	Cooperation with the student lab	Number of participants
1	Mathematics	Lower secondary level	Modul 1 (4 h) Modul 2 (3 h) Modul 3 (4 h) Implementation of materials in the classroom (4 h assumed) Modul 4 (3 h) *Total scope: 18 h*	6 to 8 weeks	Exchange of materials from the student lab Participants visit the student lab and see videos from the student lab	38
2	Biology (robotic)	Lower and higher secondary level	1 Modul (3 h) *Total scope: 3 h*	1 day	Exchange of materials from the student lab	3
3	Biology	Lower secondary level	1 Modul (3 h) *Total scope: 3 h*	1 day	Exchange of materials from the student lab	7
4	Chemistry	Lower and higher secondary level	Modul 1 (8 h) Implementation of materials created by participants in the student lab (3 ½ h) Modul 2 (3 h) *Total scope: 14 ½ h*	8 to 9 weeks	Participants visit the students lab with their own class	8
5	Physics	Lower and higher secondary level	Modul 1 (7 h) Modul 2 (4 h) Implementation of materials in the student lab (4 h) *Total scope: 15 h*	6 to 8 weeks	Participants visit the students lab with their own class	13
6	Biology (biotechnology)	Higher secondary level	Modul 1 (5 h) Implementation of materials in the student lab (6 h) Modul 2 (5 h) *Total scope: 16 h*	3 to 6 months	Exchange of materials from the student lab Participants visit the students lab with their own class	30
7	Science education	Primary level	Modul 1 (3 h) Implementation of materials in the classroom (6 h assumed) Implementation of materials in the student lab (3 h) Modul 2 (1 ½ h) *Total scope: 13 ½ h*	6 to 11 weeks	Participants visit the students lab with their own class	3
8	Science education	Primary level	Modul 1 (3 h) Modul 2 (3 h) Modul 3 (3 h) Implementation of materials in the classroom (4 h assumed) *Total scope: 13 h*	6 to 7 weeks	Exchange of materials from the student lab	3

a In North Rhine-Westphalia the primary level covers grades 1 to 4 and the secondary level is divided into lower secondary (grades 5 to 10) and higher secondary level (grades 11 to 12/13).

### 4.2 Sample

The in-service TTs took place in 2024 and 2025 and involved 105 teachers, hereafter referred to as the intervention group (IG). To provide context for the sample of teachers who registered for the in-service training, a broader single survey was conducted in 2024 among STEM educators. The comparison group (CG total) consisted of 569 teachers in total. Participants of the CG total who indicated in the survey that they have engaged in other digitalization-related TTs within the past 2 years are categorized into the subgroup CG digital. The baseline characteristics of the study participants in the IG, as well as the CG and CG subgroups are enumerated in Table 2.

**Table 2:** Baseline characteristics of the intervention group (IG) and the comparison group, including three subgroups (comparison group total [CG total]; comparison group digital [CG digital]; comparison group secondary level [CG Sec. level]; comparison group secondary level digital [CG Sec. level digital]).

Baseline characteristics	Sample	*n*	Proportion (in %)^a^	
Gender				
Female	IG	35	33.3	
	CG total	386	67.8	
	CG digital	308	68.4	
	CG sec. Level	111	42.2	
	CG sec. Level digital	99	54.4	
Male	IG	70	66.7	
	CG total	154	27.1	
	CG digital	117	26.0	
	CG sec. Level	148	56.3	
	CG sec. Level digital	80	44.0	
School level				
Primary level	IG	7	6.7	
	CG total	306	53.8	
	CG digital	268	59.6	
Secondary level	IG	98	93.3	
	CG total	262	46.0	
	CG digital	182	40.4	
		*n*	*M*	*SD*
Age in years	IG	100	41.74	9.45
	CG total	567	40.92	11.46
	CG digital	449	40.27	11.57
	CG sec. Level	263	44.81	11.24
	CG sec. Level digital	182	44.43	11.66
Teaching experience in years (without traineeship)	IG	101	11.57	7.25
	CG total	568	15.14	11.61
	CG digital	449	14.54	11.75
	CG sec. Level	262	18.77	11.43
	CG sec. Level digital	181	18.56	11.90

a Missing percentage points result from missing data points.

A comparison of the IG and CG total reveals disparities with regard to the type of school in which the participants are employed. In order to provide a more comprehensive contextual analysis of the IG, the results have been analyzed specifically in relation to participants who teach at the secondary level. Therefore, the two subgroups, CG sec. Level and CG sec. Level digital, have been formed. For the comparison group at the secondary level (CG sec. level), there were 263 participants. The final subgroup CG sec. Level digital consisted of 182 participants. Secondary level subgroups have been found to exhibit both older average ages and greater teaching experience in comparison to the overall CG.

### 4.3 Study design

**Figure 3:**
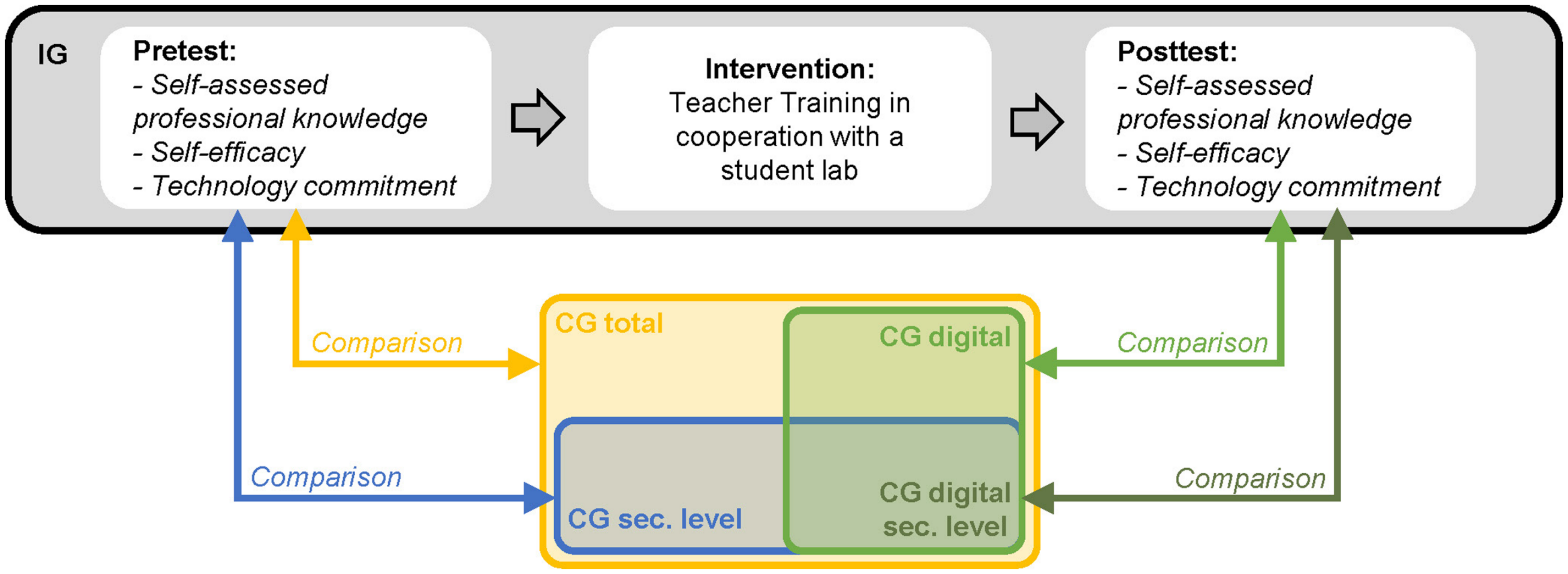
Study design: pre-posttest design within the intervention group (IG) and comparisons to the comparison groups, including three subgroups (comparison group total [CG total]; comparison group digital [CG digital]; comparison group secondary level [CG Sec. level]; comparison group secondary level digital [CG Sec. Level Digital]).

The impact of the in-service TTs was evaluated through the implementation of a quasi-experimental study design (Creswell, 2014). In a pre-posttest design, participants were asked to report on their self-assessed professional knowledge, their self-efficacy regarding the use of digital tools, and their technology commitment through a questionnaire. Between the two measurement points, participants of the IG attended one of the presented TTs (see Section 4.1). In addition, data on the same variables were collected from the CG at one measurement point in time. The IG and CG were compared in two distinct ways. The first comparison features the pretest scores of the IG with the CG total and the CG sec. Level. In the second comparison, posttest scores of the IG were examined, and a comparison was made with the CG digital and CG sec. Level digital. Figure 3 illustrates both the study design and the composition of the CGs.

### 4.4 Measurement instruments

Teachers’ self-assessed knowledge, self-efficacy, and technology commitment were measured using quantitative questionnaires. The response format consistently was a 6-point Likert scale ranging from “1 = strongly disagree” to “6 = strongly agree.” Sample items, the internal consistency, and skewness for the pre- and posttest data of the IG, as well as the CG data, are reported in Table 3. The complete scales can be found in the publications by Stinken-Rösner (2021), Beierlein et al. (2012), and Neyer et al. (2012).

**Table 3:** Sample items (translated), internal consistency and Skewness for the pretest and posttest data of the intervention group (IG) and the comparison group (CG) data of the used scales.

Scale	Sample item	*n* of items	Cronbach’s alpha (α)	Skewness
Technological Knowledge (TK)^a^	I know how to solve technical problems independently when using digital media.	4	0.87 (IG pre) 0.90 (IG post)	−0.46 (IG pre) −0.36 (IG post)
Technological Content Knowledge (TCK)^a^	I can select and use appropriate digital media to present subject content.	4	0.74 (IG pre) 0.82 (IG post)	−0.45 (IG pre) −0.53 (IG post)
Pedagogical Content Knowledge (PCK)^a^	I can help students to learn subject content in different ways.	5	0.77 (IG pre) 0.88 (IG post)	−0.16 (IG pre) −1.47 (IG post)
Technological Pedagogical Knowledge (TPK)	I can use digital media to familiarize my students with everyday examples.	4	0.78 (IG pre) 0.74 (IG post) 0.72 (CG)	−0.05 (IG pre) 0.12 (IG post) −0.50 (CG)
Technological Pedagogical Content Knowledge (TPACK)	I can design problem-centered learning environments that support students in applying and deepening subject content with the help of digital media (e.g., simulations, online materials).	5	0.90 (IG pre) 0.91 (IG post) 0.90 (CG)	−0.19 (IG pre) −0.09 (IG post) −0.03 (CG)
Technology Commitment–Technology Acceptance (TEA)	I am interested in using the latest technical equipment.	3	0.85 (IG pre) 0.87 (IG post) 0.89 (CG)	−0.19 (IG pre) −0.53 (IG post) −0.09 (CG)
Technology Commitment–Technological Competence Conviction (TEC)	I find it difficult to deal with new technology.	3	0.89 (IG pre) 0.81 (IG post) 0.90 (CG)	−1.03 (IG pre) −0.81 (IG post) 0.23 (CG)
Self-Efficacy (SE)	I can rely on my own abilities in difficult situations when using digital media in my subject.	3	0.93 (IG pre) 0.92 (IG post) 0.85 (CG)	−0.46 (IG pre) −0.48 (IG post) −0.17 (CG)

a Scales were not assessed in the CG. TK and TCK were not assessed in TT4 and TT8 of the IG.

The teachers’ self-assessed professional knowledge was measured using a questionnaire focusing on selected knowledge dimensions of the TPACK framework (Stinken-Rösner, 2021). In the IG the dimensions of TPK, PCK and TPACK were assessed. As an optional assessment tool, TCK and the general TK were additionally collected in the majority of the TTs (TT1, TT2, TT3, TT5, TT6, TT7, see Table 1). In the CG, only the TPACK and TPK dimensions were assessed, as these scales were evaluated across all TTs. The reliability of the scales ranged from 0.72 to 0.91 (Table 3). According to Taber (2018), values of α > 0.70 are considered acceptable, values of α > 0.80 are regarded as good, and values of α > 0.90 are classified as excellent. Therefore, the reliability coefficients observed in this study suggest that the scales are not only acceptable but also exhibit good to excellent internal consistency.

The degree of commitment to technology is measured using a selection of items from the short scale for measuring technology commitment (Neyer et al., 2012). Consequently, this study features a three-item scale to measure both technology acceptance (TEA) and technology competence belief (TEC) (see section 2.3). The internal consistency for both constructs is good (Table 3), similar to the findings by Neyer et al. (2012).

To measure self-efficacy regarding the domain-specific use of digital media in the classroom in accordance to each training program (SE), the general self-efficacy short scale encompassing three items (Beierlein et al., 2012) was slightly adapted. In line with results obtained during test construction by Beierlein et al. (2012), the internal consistency was good to excellent (Table 3).

### 4.5 Data analysis

Mean comparisons were conducted in order to facilitate both the assessment of measures of effectiveness and the comparison of the various samples. In the preliminary analysis, Lilliefors-corrected Kolmogorov–Smirnov tests revealed that all data were non-normally distributed. Therefore, non-parametric tests were applied.

According to Hypothesis 1, the effectiveness of the TTs was evaluated by comparing pre- and posttest mean values of the scales (TK, TCK, PCK, TPK, TPACK, TEA, TEC, and SE) for dependent groups using Wilcoxon signed-rank tests. For each construct, only participants with complete data at both the pre- and post-measurement times were included in the respective analyses (pairwise deletion). The interpretation of effect sizes followed the convention established by Blanz (2015), whereby values greater than 0.1 are designated as weak, values between 0.3 and 0.5 are classified as moderate, and values greater than 0.5 are labeled as strong.

In order to compare the teachers’ TPK, TPACK, TEA, TEC, and SE between the independent groups (namely the IG and CGs), Mann–Whitney U tests have been used. The pretest scores of the IG were compared with the data from the CG total and CG sec. Level group (according to Hypothesis 2.1). The posttest values of the IG were compared with the data from the CG digital and CG sec. Level digital, that is, with teachers who took part in other digitization-related training programs that were not part of the project (according to Hypothesis 2.2).

## 5 Results

### 5.1 Impact of the in-service teacher trainings

**Figure 4:**
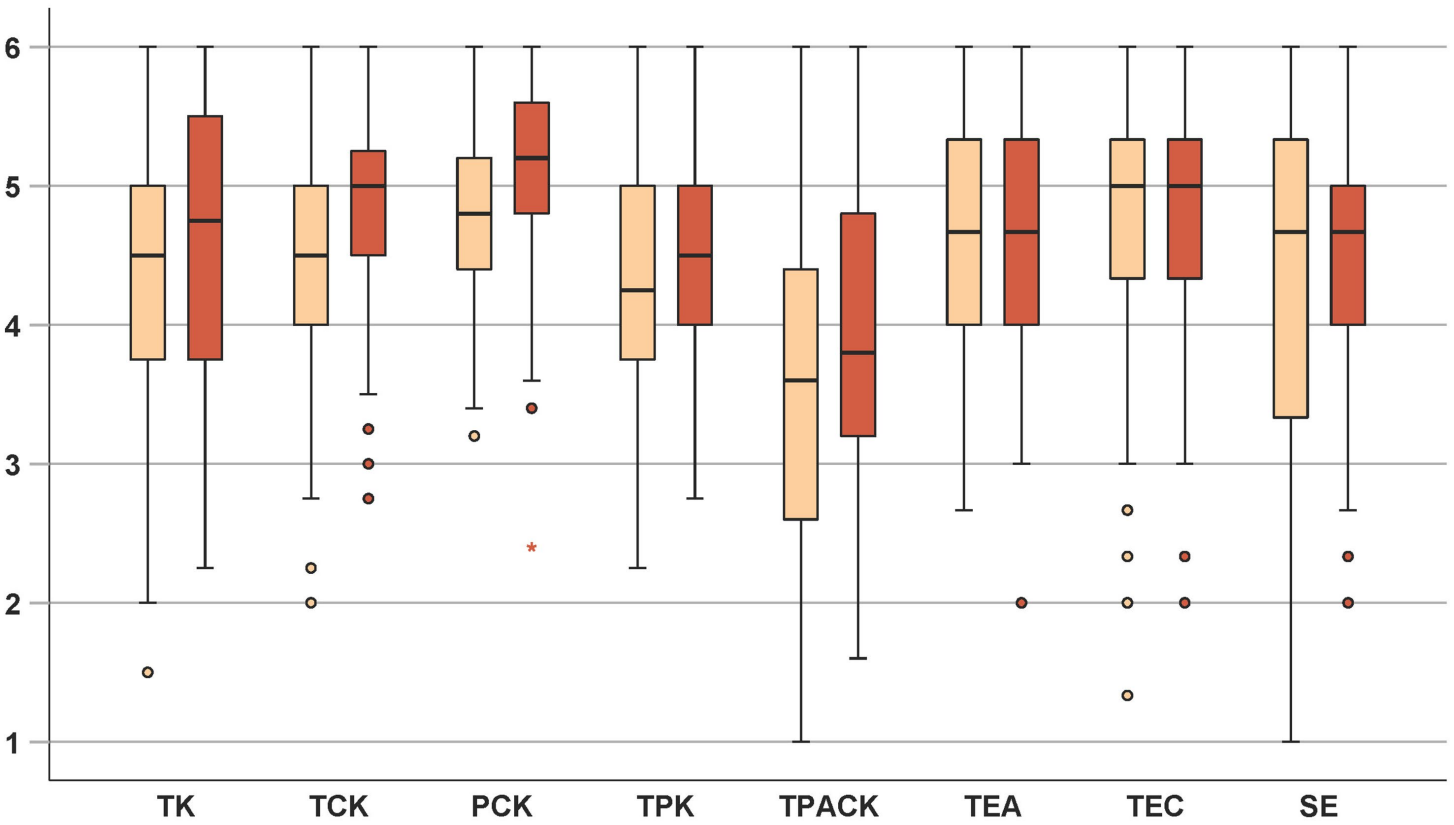
Boxplots for pretest data (left, light orange) and posttest data (right, dark orange).

Descriptive statistical data of the pre- and posttest, along with the results of the mean comparisons, are presented in Table 4 and Figure 4. In the pre-post comparison (according to RQ1, Hypothesis 1) changes in the professional knowledge dimensions of TK, TCK, PCK, TPK, and, TPACK were found. Weak effects were found in the dimensions of TK and TPK and moderate effects were evident in the areas of TCK and TPACK. A strong effect was observed in the PCK dimension. While the descriptive results for TEA, TEC, and SE show an increase, no significant differences between the pretest and posttest were found for these measures.

**Table 4:** Descriptive statistics and mean comparison of the pre- and posttest.

Variables		*n*	*M*	*SD*	Wilcoxon test			
					*z*	*p*	*r*	*df*
TK	Pre	71	4.45	0.93	2.37	0.018	0.28	70
	Post	71	4.62	1.03				
TCK	Pre	71	4.56	0.81	3.38	<0.001	0.40	70
	Post	71	4.89	0.77				
PCK	Pre	82	4.78	0.59	5.00	<0.001	0.55	81
	Post	82	5.12	0.71				
TPK	Pre	81	4.28	0.86	2.36	0.018	0.26	80
	Post	81	4.49	0.78				
TPACK	Pre	81	3.50	1.20	3.26	0.001	0.36	80
	Post	81	3.88	1.11				
TEA	Pre	82	4.58	0.94	1.13	0.260	0.12	81
	Post	82	4.66	0.88				
TEC	Pre	82	4.75	1.04	−0.08	0.940	−0.01	81
	Post	82	4.76	0.95				
SE	Pre	82	4.34	1.25	1.17	0.242	0.13	80
	Post	82	4.49	1.06				

The boxplots in Figure 4 illustrates the development of the constructs from pretest to posttest. In the knowledge-related dimensions (TK, TCK, PCK, TPK, TPACK), the median values increased from the pre- to the posttest. For the constructs related to technology commitment and self-efficacy (TEA, TEC, SE) the median remains stable. Across all constructs, the maximum scale value (6) is observed in both measurement points. With regard to the distribution of data, a general reduction in dispersion toward the lower end of the scale is evident in the posttest, as indicated by the shorter whiskers observed in most constructs. This pattern is not observed for TEC, where the spread remains largely unchanged.

### 5.2 Comparisons between the intervention group and comparison groups

According to RQ2.1 and RQ2.2, the results of the comparison between IG and CGs are presented in Tables 5, 6.

**Table 5:** Comparison between the intervention group (IG) in the pretest and the comparison group in total (CG total) and the teachers of secondary level (CG sec. level).

Variable	Group	*n*	*M*	*SD*	Mann–Whitney-U-Test			
					*U*	*z*	*p*	*r*
TPK	IG	104	4.35	0.85				
	CG total	566	3.48	0.85	44,628	8.43	<0.001	0.33
	CG sec. Level	263	3.46	0.84	20,913	7.95	<0.001	0.41
TPACK	IG	104	3.55	1.18				
	CG total	560	3.19	1.00	34,565	3.04	0.002	0.12
	CG sec. Level	261	3.16	1.02	16,237	2.93	0.003	0.15
TEA	IG	104	4.69	0.94				
	CG total	566	3.02	1.14	50,537	11.68	<0.001	0.45
	CG sec. Level	262	2.93	1.16	23,707	11.09	<0.001	0.58
TEC	IG	104	4.75	1.05				
	CG total	566	3.57	1.10	45,954	9.16	<0.001	0.35
	CG sec. Level	262	3.56	1.06	21,496	8.66	<0.001	0.45
SE	IG	104	4.36	1.20				
	CG total	559	3.19	1.06	44,356	8.56	<0.001	0.33
	CG sec. Level	258	3.17	1.07	20,541	7.94	<0.001	0.42

**Table 6:** Comparison between the intervention group (IG) in the posttest and the participants of the comparison group who have visited a digitalization-related teacher trainings in the past 24 months (CG digital) and the teachers of secondary level (CG sec. level digital).

Variable	Group	*n*	*M*	*SD*	Mann–Whitney-U-Test			
					*U*	*z*	*p*	*r*
TPK	IG	84	4.53	0.79				
	CG digital	448	3.49	0.88	30,168	8.85	<0.001	0.38
	CG sec. Level digital	182	3.47	0.89	12,305	8.05	<0.001	0.49
TPACK	IG	84	3.91	1.10				
	CG digital	445	3.29	1.01	24,615	4.62	<0.001	0.20
	CG sec. Level digital	181	3.33	1.05	9,805	3.80	<0.001	0.23
TEA	IG	85	4.69	0.88				
	CG digital	448	3.01	1.13	33,243	10.95	<0.001	0.47
	CG sec. Level digital	181	2.95	1.14	13,566	10.08	<0.001	0.62
TEC	IG	85	4.77	0.94				
	CG digital	448	3.61	1.10	29,915	8.41	<0.001	0.36
	CG sec. Level digital	181	3.57	1.07	12,255	7.85	<0.001	0.48
SE	IG	84	4.50	1.04				
	CG digital	445	3.24	1.06	29,749	8.65	<0.001	0.38
	CG sec. Level digital	179	3.26	1.08	11,871	7.60	<0.001	0.47

According to Hypothesis 2.1, significant differences were identified in TPK, TPACK, technology acceptance (TEA), technology competence belief (TEC), and self-efficacy (SE) between the IG and the CG total. Furthermore, a similar discrepancy was observed between the IG and the CG secondary level. The effects are moderate in the areas of TPK, TEA, TEC, and SE, whereas there was a strong difference in TEA between the IG and the CG secondary level and weak differences were found in TPACK. The effects in comparison with the secondary level CG tend to be higher in all areas than in comparison with the overall CG.

According to Hypothesis 2.2, comparisons with the digitalization-trained CG reveal a similar pattern in the dimensions of TPK, TPACK, TEA, TEC, and SE (see Table 6): In this case, posttest results of the IG are significantly higher than the results of the CG digital as well as the CG sec. Level digital in all analyzed areas. In the domain of TPACK, the observed effects are found to be weak, while the effects in TPK, TEA, TEC, and SE are assessed as moderate. Furthermore, a strong effect is observed between the groups in TEA, when compared with the CG digital secondary level. The effects observed in the comparison of the IG with the CG sec. Level digital were found to be more pronounced compared to those reported in the comparison of the IG with the CG digital.

## 6 Discussion

According to Hypothesis 1, participation in a domain-specific TT in cooperation with student labs is expected to lead to an increase in self-assessed professional knowledge, self-efficacy, and technology commitment. However, results can only partially confirm this hypothesis. While a significant increase was identified from pre- to post-data for various dimensions of self-assessed professional knowledge (according to the TPACK framework), no such increase was observed concerning self-efficacy, technology acceptance, and technological competence beliefs (both as dimensions of technology commitment). For the latter constructs, values remained at the same level as before participating in the TTs.

The increase in self-assessed professional knowledge aligns well with the aims of the TTs, which were specifically designed to teach digitalization-related skills and the use of domain-specific media. Participating teachers hence had to deal with the technical basics of domain-specific media and thus expand their technical knowledge (TK) as well as content-specific technological knowledge (TCK). Teachers needed to consider how media can be integrated into lessons in a pedagogically meaningful way (TPK) to teach domain-specific content effectively (TPACK). In addition, the program content is linked to domain-specific pedagogical principles, which has a positive effect on domain-specific pedagogical content knowledge (PCK). Results suggest that the TTs tend to have a positive effect on all TPACK dimensions, although the effect sizes do vary. Moderate (TK, TPK, TCK, TPACK) and large effect sizes (PCK) were measured (see Table 4). The greatest effect sizes measured for PCK, TCK, and TPACK do align with the domain-specificity of the TTs designed in this study, as hypothesized in Hypothesis 1. This also holds true for the low effect sizes measured for TK and TPK: Both knowledge dimensions crosscut the disciplines and are only indirectly addressed by the TTs, fitting the lower effect sizes measured. In accordance with other studies, which have shown an increase in all TPACK dimensions of both pre-service (Stinken-Rösner et al., 2023) and in-service (Lehiste, 2015) teachers, our results also show that an increase of self-assessed knowledge in the TPACK dimensions can be achieved by participating in digitalization-related domain-specific TTs.

The pre- and post-data of self-efficacy (SE), technology acceptance (TEA) and technology competence beliefs (TEC; both as dimensions of technology commitment) reveal no significant differences, although the data indicate low increases of the means (see Table 4). In line with other studies, such as Runge et al. (2024), no impact of TT on self-efficacy was found. Notably, teachers had high initial values regarding these constructs before registering for the TT (see below for a comparison with the CG). The frequent occurrence of the maximum value (6) in both measuring points (see Figure 4) and the negative skewness of the distributions (see Table 3) suggest that ceiling effects may be a contributing factor. Furthermore, it is possible that not all participants were able to implement content from the TT immediately during the implementation phase in the classroom, depending on the point of reference and the topics covered in their current school lessons. While the implementation of materials in the student lab allows teachers to integrate them into a specific and organized instructional setting, it is not necessarily linked to regular classroom instruction. This might have contributed to the fact, that participants’ self-assessments remained similar in comparison to their initial evaluations at the beginning of the TT.

Hypothesis 2.1 expects participating teachers to already have more self-assessed professional knowledge, a more pronounced self-efficacy and a higher technology commitment than other teachers in STEM education before enrolling in the TTs. This hypothesis was confirmed, as the teachers who took part in the conceptualized training courses had significantly higher values in the above-mentioned constructs (see Table 6). Results indicate that those teachers, who already had more knowledge and experience in the field of digitalization, decided to participate. Consequently, TTs of this study did not attract teachers with limited levels of professional knowledge, less pronounced self-efficacy and limited technology commitment. One potential explanation for this participation pattern may be found in differential inclination toward TT engagement. Desimone et al. (2006) found that teachers with stronger content knowledge were significantly more likely to participate in sustained professional development, suggesting that subjective inclination rather than objective need primarily drives participation decisions. Teachers with lower technological commitment may be particularly reluctant to voluntarily invest additional time in digital media, perceiving it as extra work. Therefore, future studies should consider how to specifically target and motivate these teachers.

Finally, Hypothesis 2.2 states that participants would feature similar levels of professional knowledge, self-efficacy and technology commitment after participating in the designed TTs compared to teachers who have participated in other, previous TTs on digitalization-related topics. The results of the study suggest that this hypothesis cannot be confirmed. The participating teachers show significantly higher values than those who have taken part in previous other digitalization-related TTs (see Table 6). One possible explanation for this result could be a higher effectiveness of the designed TTs in the IG, leading to a greater development in teachers’ professional knowledge, self-efficacy and technology commitment. Differences between the TTs in which the IG and CG participated cannot be analyzed because the concepts of the TTs in which the CG participated are unknown. The observed discrepancy in outcomes might be attributed to the varying temporal points at which the surveys were conducted. The IG was asked to complete the posttest directly after participating in the TT, while the CG was surveyed about their participation in digitalization-related further TT at intervals of 24 months. There are no findings to suggest whether the teachers in the CG would have indicated higher values if they had been asked immediately after participating in the TT. It is possible that these teachers have had contrary experiences since then, potentially leading to a relativization of these values.

### 6.1 Limitation

When considering the results, certain limitations of the current study must be taken into account, which may restrict the generalizability of the findings. Firstly, it should be noted that the sample size of the individual TTs constitutes a key limitation of this study. Some TTs have a high number of participants (*n*_TT1_ = 38; *n*_TT6_ = 30), while others only have a small number of participants (*n*_TT7_ = 3; *n*_TT8_ = 3; *n*_TT2_ = 3; *n*_TT3_ = 7). Consequently, an in-depth comparison of the results between the various TTs, as well as between the modular and shortened TTs, was not possible.

Secondly, the aspect of small sample sizes was already an effect during implementation of individual TTs. The TTs on which the study was based were each designed modularly. For some TTs, no participating teachers could initially be acquired. It was assumed that the teachers would not be able to take time off from their school due to the spacing of the modular training days. These TTs were therefore rescheduled as one-time TTs with the intention of attracting teachers. This approach resulted in a slight increase in the number of participants. However, as the modular structure for these training courses was removed, their results can only be compared with those of other TTs to a limited extent.

Thirdly, a central limitation of the study concerns the different structures of the TTs. The analyzed trainings differ in terms of duration, used learning materials and addressed types of school. As there was no standardized concept, the results cannot simply be transferred to similar TTs. Due to the structural differences between the TTs, it is not possible to clearly determine which specific characteristics contributed to the observed effects.

Fourthly, the teachers’ professional knowledge was investigated using self-assessments. This leads to limitations in determining the actual level or acquisition of knowledge. As self-assessed competence may not fully correspond to actual competence (Ernst et al., 2025), future studies may investigate teachers’ actual professional knowledge.

### 6.2 Further research

It is recommended that future research address the limitations observed in the current study. It is notable that assessment measures taken immediately after TTs often reveal more positive effects than those collected at later dates. This may be indicative of the fact that the improvements observed in the IG are not stable over time. In order to elucidate this issue, it is recommended that future studies employ longitudinal designs in order to capture the durability of these effects. Furthermore, it is recommended that larger sample sizes are utilized in order to enhance both statistical power and the generalizability of the findings. To better determine causality and further investigate individual effects, a controlled design should be employees. In addition, a domain-specific perspective is warranted. Comparisons between modular versus shortened teacher training formats, as well as the implementation of trainings within a collegial framework, should be explored. This approach has the potential to enhance the engagement of teachers who genuinely require professional development, particularly those with lower professional knowledge and technological commitment, who may otherwise perceive digital media integration as an additional burden. Furthermore, future research studies are recommended to examine the impact of cooperation with student labs in more detail. This should involve systematically comparing the implementation phases in student labs with those in standard classrooms.

Finally, data collection of the CG differs in certain aspects from data collection of the IG, which limits the comparability of the results between the two groups. Data collection of the CG was not carried out as part of this study, but by external partners from other universities who are also participating in the joint project LFB-Labs-digital. For this purpose, a questionnaire was developed that includes various constructs (TEA, TEC, TPK, TPACK, and SE are relevant for the current study) as well as demographic data. The questionnaire was sent to teachers of various subjects and school types with the request to complete it. Measuring TPACK has been limited to the two constructs with the largest loadings (TPK and TPACK). As a result, a comparison of professional knowledge can only be made in the TPK and TPACK dimensions. Furthermore, data collection differed in terms of the timing of the survey. While the teachers in the IG were surveyed directly before the training or at the beginning of the training, the CG was surveyed retrospectively. They were asked to state whether they had taken part in a TT in the past 24 months and whether this was related to digitalization. It can therefore be assumed that sustainability effects had an influence on the measured values. Furthermore, it is unclear how the content and methodology of the TTs attended by the teachers in the CG were specifically designed. While the TTs in this study specifically addressed domain-specific digitalization-related skills, it is possible that the teachers in the CG attended TTs that addressed general digitalization-related skills. This study does not cover this topic through the questionnaires used. Additionally, due to the absence of a controlled design in the current study, it is difficult to isolate the specific effects of individual intervention components.

## 7 Conclusion

In conclusion, the results of the present study suggest that domain-specific modular TTs in cooperation with student labs are effective in promoting digitalization-related skills among in-service teachers. Results indicate, that participating in such TTs significantly improves teachers’ self-assessed professional knowledge. Therefore, participation in TT can be seen as a way of supporting teachers’ professional development, which could positively impact students’ learning processes. In this context, student labs play a particularly important role. Teachers profited from student labs as an environment where they could test innovative, domain-specific digital media and learning settings. An increase in self-efficacy and technology commitment was expected as a result. However, participating in the TTs did not change the means significantly, hence the effectiveness of the TTs in this regard could not be proven. Nevertheless, the implementation phase as one characteristic of effective TTs is an important part of the transfer process of digital, domain-specific media into teaching practice. Therefore, future studies should examine this aspect more closely and consider it in the design of future TTs. The findings show that the targeted linking of modularized TT formats with classroom-like settings such as student labs is a promising way to effectively strengthen the digital skills of teachers and thus support educational processes in the digital transformation in the long term.

## Data Availability

The datasets presented in this article are not readily available because this study is part of ongoing research projects and qualification works in which further analyses are planned. Requests to access the datasets should be directed to Anna Reher, anna.reher@uni-bielefeld.de and Mathias Ziegler, mziegler@physik.uni-bielefeld.de.
